# Healthcare services provided to pregnant women with HIV and their outcomes at primary healthcare clinics in the Free State province, South Africa

**DOI:** 10.4102/sajhivmed.v27i1.1792

**Published:** 2026-02-28

**Authors:** Olive P. Khaliq, Ahmad Jassen, Nomakhuwa E. Tabane, Jagidesa Moodley

**Affiliations:** 1 Department of Paediatrics and Child Health, Faculty of Health Sciences, University of the Free State, Bloemfontein, South Africa; 2 Department of Obstetrics and Gynaecology, College of Health Sciences, University of KwaZulu-Natal, Durban, South Africa

**Keywords:** HIV, pregnancy, testing, management, treatment, neonates

## Abstract

**Background:**

HIV prevalence among pregnant women in South Africa was very high at 25.3% of infections reported in 2022. KwaZulu-Natal province had the highest HIV prevalence of 34.2%, followed by the Eastern Cape with 32.0% infections, and the Free State with a prevalence of 28.8%.

**Objectives:**

To determine the HIV prevalence and healthcare services provided to pregnant women with HIV at primary healthcare clinics in the Free State province.

**Method:**

This was a retrospective evaluation of all antenatal records from 2020 to 2023 at primary healthcare facilities in the Free State province, South Africa. All pregnant women who started antenatal care and delivered at the clinic were included in the study. Maternal demographic and clinical data, including HIV status, the clinical management of HIV, and perinatal outcomes were recorded. Maternal records of unbooked mothers and those who did not deliver at the clinic were excluded.

**Results:**

The antenatal records of 668 pregnant women during the period 2020–2023 were reviewed. The prevalence of HIV was 27.9%, of which 22.4% tested for the first time at booking. Among pregnant women living with HIV, 4.2% had a CD4-count of < 200 cells/mm^3^, 48.7% had no viral load recorded and 85% were on antiretroviral therapy. Only 69.6% of the HIV-negative women were retested. All infants (*n* = 187) born to mothers living with HIV had a negative HIV birth polymerase chain reaction test. Approximately 41% of the HIV-exposed infants had a low-birth weight. In addition, two low-birthweight infants were stillbirths.

**Conclusion:**

HIV prevalence among pregnant women remains high, with gaps in viral load monitoring and HIV retesting and early antenatal booking. Low-birth-weight rates were higher among HIV-exposed infants, indicating ongoing vulnerability despite available services.

**What this study adds:** The prevalence of HIV remains high at primary healthcare clinics. Gaps in care include late booking, poor viral load monitoring, and low retesting rates among HIV-negative women. Low birth weight among HIV-exposed infants highlights the need to improve maternal and child healthcare outcomes.

## Introduction

The HIV pandemic is still a global burden as infection rates are increasing, according to the WHO. About 40.8 million people were living with HIV in 2024.^1^ Approximately 45% of people living with HIV were women and girls.^2^ In 2024, 63% of new cases of HIV-positive women and girls were recorded in sub-Saharan Africa.^2^ In South Africa, the number of people living with HIV remains high, with 7.7 million in total and 4.9 million being women.^3,4^ The WHO reported in 2023 that 264 889 pregnant HIV-positive women were receiving antiretroviral therapy (ART), which reduces the risk of vertical transmission from mother to child.^5^ In 2022, 25.3% of pregnant women in South Africa were reported to be living with HIV, indicating that the infection rates are still high in this group.^6^ In 2022, KwaZulu-Natal province had an HIV prevalence of 34.2%, followed by the Eastern Cape with 32.0% infections, and the Free State with 28.8%.^6^ Women aged 20–24 years had a higher prevalence of HIV (16.4%) than those in other age groups (15–24 years, 13.6%; and 15–19 years, 7.6%).^6^

Pregnant women living with HIV are at a risk of adverse pregnancy outcomes, including preterm births, low-birth-weight babies, small-for-gestational-age babies, and stillbirths. Furthermore, poor adherence to ART or no ART uptake can lead to vertical transmission of HIV.^7,8,9^ For women who are already living with HIV and on ART, it is recommended that treatment is continuous during pregnancy, childbirth, and the postpartum breastfeeding period. HIV testing is not mandatory, but is recommended.^10^ All newly HIV-diagnosed pregnant women should be offered ART, regardless of their gestational age, CD4 count, or clinical stage. To monitor the newly diagnosed women, a viral load test should be performed after 3 months of ART initiation. For women living with HIV, viral load measurement should be done at the first antenatal care (ANC) visit, and if < 50 copies/mL, viral load should be measured again at delivery.^11^

Antenatal care is important for the comprehensive treatment and management of HIV according to the guidelines for vertical transmission prevention.^12^ Pregnant women are expected to receive ANC as recommended by the WHO.^7^ The guidelines state that all pregnant women should be offered an HIV test at the first ANC visit, and that a retest in HIV-negative mothers should be performed routinely at every ANC visit, which is monthly Basic Antenatal Care (BANC) Plus national guidelines.^13,14^

In South Africa, 31.6% of women between the ages of 20 and 34 years are living with HIV, and one in three women attending ANC is living with HIV.^11,15^ Moreover, women who are HIV positive for the first time during pregnancy are reported to be at a risk of ‘loss to follow-up’ and therefore add to difficulties in ensuring continuation of treatment of HIV post delivery.^16,17,18,19^ Furthermore, HIV is one of the leading causes of maternal deaths in South African women aged 15–44 years.^20^ According to Clouse et al.,^15^ prenatal care at a primary care clinic, at a hospital or at other approved obstetric facilities, and a return to regular HIV care at the primary care clinic, are both parts of a broken continuum of care in South Africa.^16^ To accurately describe the HIV continuum of care for expectant mothers and assess the efficacy of national ART programmes in treating expectant and postpartum women, improved pregnancy-related data systems are required.^15^ Therefore, this study aims to assess the prevalence and management of HIV in pregnant women at two primary healthcare clinics in the Free State province of South Africa.

## Research methods and design

This was a cross-sectional retrospective study conducted at two primary healthcare facilities in the Free State province of South Africa. Data were collected by a research nurse – who is a qualified midwife – from the maternity case records of those who attended antenatal clinics from January 2020 to May 2023, regardless of their HIV status. Data were then captured on RedCap^®^ (Research Electronic Data Capture) at the University of the Free State,^21^ and analysed by a biostatistician. The study sites were chosen based on factors such as size, location, and the number of pregnant women who visited the clinics. The study clinics were chosen because they had a high volume of antenatal visits each day and their location was central, making them easily accessible. Before the study was initiated, ethical and provincial health authority approvals were obtained (UFSHSD2022/1399/2803-0001). All pregnant women who started ANC and delivered at the clinic were included in the study. Maternal demographic data, maternal history, clinical data, HIV status, and HIV-related adverse outcomes of both mothers and babies were recorded. Missing data were included as ‘not recorded’ as long as the delivery occurred at the clinic, in order to identify areas that need to be improved for better care at the clinics. Maternal records of unbooked mothers and mothers who did not complete their ANC at the clinic were excluded.

Gravidity was defined as the number of times a woman has been pregnant, including the current pregnancy, while parity was defined as the number of times a woman has delivered a live baby, but excluded the current pregnancy.^22^ Haemoglobin levels vary with different trimesters of pregnancy. The WHO categorised these levels as follows: < 11 g/dL in the first and third trimester, and < 10.5 g/dL in the second trimester, and recommends that any levels below these cutoffs warrant a full blood count assessment.^23^ In this study, haemoglobin levels were categorised into two groups (< 10 g/dL and > 10 g/dL) at booking.

For this study, live birth was defined as the full expulsion or extraction of a product of conception from its mother, regardless of the length of the pregnancy, that, following such separation, breathes or exhibits any other indication of life, such as heartbeat, umbilical cord pulsation, or distinct voluntary muscle movement, regardless of whether the placenta is attached or the umbilical cord has been severed.^24^

### Statistical analysis

Data were analysed descriptively. The mean and standard deviation or median and interquartile range were used to summarise the continuous variables. Frequencies and percentages were used to describe the categorical variables.

### Ethical considerations

Ethical clearance to conduct this study was obtained from the University of the Free State, Health Sciences Research Ethics Committee (reference number: UFS-HSD2022/1399/2803-0004).

## Results

### Demographics and obstetric history

A total of 668 maternal records were included in the study. The median maternal age was 25 years, interquartile range (IQR: 21–30). The majority of the pregnant women were between the ages of 20 and 35 years, *n* = 559/668 (83%). Teenage pregnancies occurred in 93/668 (13.9%) of the women. Most women had a gravidity of 2–5 (*n* = 456, 66.2%) and a parity of 1–5, *n* = 442/668, (67.3%). Approximately 42/668 (6.3%) had previous intrauterine foetal death, *n* = 2/668 (0.4%) had previous preterm births, and *n* = 7/668 (1%) had previously terminated their pregnancies by choice (Table 1).

**TABLE 1:** Maternal demographics and clinal investigations at antenatal care (*N* = 668).

Maternal characteristics	*n*	%
**Maternal age (years)**		
≤ 19	93	13.9
20–35	559	83.0
≥ 36	16	2.4
**Obstetric history**		
Gravidity		
1	210	31.4
2–5	456	66.2
≥ 6	1	0.1
Not recorded	1	0.1
Parity		
0	225	33.7
1–5	442	67.3
Not recorded	1	0.2
**Previous birth outcomes**		
Intrauterine foetal death		
Yes	42	6.3
No	626	93.7
**Preterm birth**		
Yes	2	0.4
No	659	98.6
Choice of termination of pregnancy	7	1.0
**Antenatal care**		
Gestational age at first antenatal visit (weeks)		
< 20	213	31.9
> 20	341	68.1
**Screening tests**		
Ward haemoglobin (g/dL)		
≤ 10 g/dL	86	12.9
≥ 10 g/dL	483	77.2
Not recorded	99	14.8
Full blood count done (haemoglobin < 10 g/dL)		
Yes	29	33.7
No	57	66.3

Note: Maternal age (years): Median 25 years old; interquartile range (quartile 1 – quartile 3) 21–30 years old.

### Antenatal care

A large number of pregnant women (*n* = 341/668, 68.1%) had late booking (> 20 weeks of gestation), and 213/668, (31.9%) booked on time (< 20 weeks of gestation). Haemoglobin levels were measured in all women, and 86/668 (12.9%) had levels < 10 g/dL. Full blood counts were only measured in 57/668 (66.3%) of these women.

In this study population, 187/668 (27.9%) of the pregnant women were living with HIV, of which 42/187 (22.4%) did not know of their status at booking; 145/187 had a known HIV status. The CD4 count was ≤ 200 cells/mm^3^ in 8/187 (4.2%) of women, including the 22.4% that tested for the first time at booking, while a viral load of ≤ 50 copies/mL was recorded in 67/187 (35.8%) of all women living with HIV. Of women living with HIV, 91 out of 187 (48.7%) had no viral load recorded.

### HIV treatment

Of the women with a known HIV status, 159/187, (85%) were on ART. Those who only knew of their status at their first antenatal booking were started on ART immediately, regardless of their CD4 counts.

### HIV retesting

Of the 668 pregnant women, 481 tested negative for HIV at booking; only 335/481 (69.6%) were retested. Retesting was done a number of times during the different trimesters. In 71/335 cases (21.2%), HIV retesting was performed once during the second trimester, once in the third trimester in 143/335, (42.7%), and in some cases, twice in the third trimester (*n* = 10/335, 2.9%). One woman was retested four times (once in the second trimester, twice in the third, and once during labour). One woman was tested once during labour (Table 2). Only one woman seroconverted following a repeat test in the second trimester.

**TABLE 2:** The management of HIV in pregnant women in the Free State province, 2020–2023 (*N* = 668).

Clinical parameters	*n*	%
**HIV status**		
Total number reactive	187	27.9
Known HIV status (reactive)	145	77.5
Newly tested (reactive)	42	22.4
CD4 count (cells/mm^3^)		
< 200	8	4.2
≥ 200	93	49.7
Not recorded	86	45.9
Viral load at booking (copies/mL)		
< 50	67	35.8
≥ 50	29	15.5
Not recorded	91	48.7
HIV treatment (HAART) at booking		
Yes	159	85.0
No	28	15.0
HIV retest done when the first test was negative?		
Yes	335	69.6
No	146	30.4
Gestational age intervals for HIV retesting (*n* = 335)		
Once in second trimester	71	21.2
Once in third trimester	143	42.7
Once in second trimester and once in third trimester (two times)	57	11.0
Twice in third trimester	10	2.9
Once in third trimester and once in labour (two times)	4	1.2
Once in second trimester and twice in third trimester (three times)	3	0.9
Twice in third trimester and once in labour (three times)	5	1.5
Once in second trimester, twice in third trimester and once in labour (four times)	1	0.3
Once in labour	1	0.3
**Infant outcomes**		
Baby weight (g)		
1000–1499	1	0.1
1500–2499	43	6.4
HIV exposed	18	41.0
> 2500	624	93.4
Stillbirths		
HIV exposed	1	0.1
HIV unexposed	1	0.1
Preterm births		
HIV exposed	6	0.9
HIV unexposed	13	1.9
Congenital abnormalities		
Dysmorphic features	1	0.1
Abdomen hepatosplenomegaly	1	0.1
None	666	99.3

HAART, highly active antiretroviral therapy.

### Infant outcomes

Forty-four out of 668 (6.5%) of the infants had low birth weight. Eighteen out of 668 (41%) of these infants were HIV exposed, but none of them were HIV positive on polymerase chain reaction (PCR) testing done at birth. Of the 28 mothers who were living with HIV but not on ART, 7/28 of their infants had low birthweight. One infant out of 668 with very low birthweight and hepatosplenomegaly, was born to a mother living with HIV on ART. Two macerated low-birthweight stillbirths occurred out of the 668 infants and both had congenital abnormalities (Table 2).

## Discussion

The study found that the HIV prevalence among pregnant women attending two primary healthcare clinics (*N* = 668) in the Free State province was high at 27.9%, with nearly one-quarter of women living with HIV being newly diagnosed during pregnancy (22.4%). While 85% of women living with HIV were on ART, nearly half had no recorded viral load, limiting assessment of treatment effectiveness. Additionally, most women booked late for ANC (68.1%). HIV-negative women were not consistently retested, with 30.4% missing recommended repeat testing. Low-birth-weight infants accounted for 6.5% of births, and 41% of these were HIV-exposed, although no cases of vertical transmission were detected.

Most women (83%) were within the 20–30 year old age group, which is in keeping with a South African report that 70% of child births occurred in women between the ages of 20–35 years in 2023.^25^ Approximately 13.9% of the pregnant women in the study were teenagers. This is lower than the overall prevalence of 39% in sub-Saharan Africa. Factors associated with teenage pregnancies include child marriages,^26^ low levels of education, poor socioeconomic status,^27^ and incorrect use of contraceptives.^28,29,30^

The majority of pregnant women in this study booked late for ANC, after 20 weeks of gestation. This is higher than the rates reported globally (58.6%),^31^ but lower than a study from Limpopo that found 84% of pregnant women who initiated late ANC.^32^ Tukisi et al. conducted a study on factors influencing late antenatal booking from Tshwane district in South Africa, and mentioned that some of the factors included complications in previous pregnancies, long waiting hours at the antenatal clinic, and the unpleasant attitude of midwives.^33^ In this study, the reasons for late booking are unknown, as this was a retrospective study. Delayed initiation of ANC may reduce opportunities for early risk identification, timely management of complications, and provision of preventive interventions such as nutritional supplementation and screening for infections such as HIV. According to the 2016 Department of Health guidelines for maternity care, women should have their first ANC visit as soon as she suspects that she might be pregnant, or after her first missed period.^34^

The prevalence of HIV in the study was 27.9%. These results correspond with the national prevalence of 27.5%, reported by the Antenatal HIV sentinel survey in 2022.^6^ Provincially, KwaZulu-Natal accounted for the highest prevalence of HIV (37.2%), the Northern Cape (15.2%) and the Western Cape (16.3%) were the lowest.^6^ Of concern is that 22.4% of women living with HIV were newly diagnosed during pregnancy, as this is higher than a study conducted in the Western Cape, South Africa, which found a prevalence of 1.3% (11/828) in pregnant women.^35^ The current study highlights the importance of HIV testing preconception and early ANC in pregnant women for early diagnosis and management of HIV to prevent mother-to-child transmission.

The majority (85%) of pregnant women living with HIV were on ART, consistent with national and international recommendations.^12,36^ According to the 2025 Joint United Nations Programme on HIV/AIDS Fact Sheet, 84% of pregnant women are on ART globally.^37^ In South Africa, 93% of pregnant women living with HIV are on ART.^37^ According to the South African HIV Clinicians Society (SAHCS) guidelines for ART, there is a 40% risk of HIV vertical transmission from mother to child if HIV is not managed during pregnancy. There is a 5% risk of transmission during pregnancy, 15% – 20% during delivery, and 20% during breastfeeding.^38^ In this study, only 35.8% achieved viral suppression (≤ 50 copies/mL). Viral loads (VL) > 50 copies/mL increase the risk of vertical transmission; therefore, VL should be measured following 3 months of ART and at delivery.^38^

Only 69.6% of HIV-negative women underwent repeat testing. This is concerning, as retesting during pregnancy and at delivery is recommended in high-prevalence settings to detect seroconversions. The fact that only one woman (0.3%) seroconverted in this cohort may reflect low incidence, but also highlights the potential risk of missed diagnoses in those not retested. Our findings are consistent with a recent Ugandan study that found an 85% retesting rate. Factors associated with retesting included secondary education and tertiary education, partner support, and regular antenatal visits.^39^ In another study in Mozambique, 84% of 28 233 pregnant women were retested for HIV at an antenatal clinic, and 26 of the women seroconverted.^40^ According to the Guideline for the Prevention of Mother to Child Transmission of Communicable Infections, South Africa, all pregnant women who tested negative for HIV should be retested at every routine visit.^41^ However, in this study, 30.4% of the pregnant women were not retested.

Birth outcomes revealed that 6.5% of infants were of low birth weight, of which 41% were HIV-exposed. Furthermore, none of the infants born to mothers living with HIV had a positive birth PCR, reflecting the effectiveness of ART in preventing vertical transmission, despite gaps in viral load monitoring. Approximately 28 out of 187 (15%) of pregnant women living with HIV in our study were not on ART, and 25% of their babies had low birth weight. The reason why these women were not on ART is unknown. SAHCS 2023 guidelines recommend that all pregnant and breastfeeding women should be on lifelong ART.^38^ Fleșeriu et al. also found a significant difference in maternal outcomes of HIV-exposed babies versus non-exposed babies. Significant differences were found in birthweight (*P* < 0.001), and length (*P* < 0.001).^42^ One of the HIV-exposed babies was stillborn, and one was born with hepatosplenomegaly in the current study. HIV-exposed babies have higher morbidity compared to HIV-unexposed babies, with gastrointestinal and respiratory infections as the major causes of morbidity.^43^ Furthermore, 30% of HIV-exposed babies have complications compared to HIV-unexposed babies, even if they are HIV negative, and 20% have more frequent hospital admissions compared to HIV-unexposed babies.^44^

### Strengths and limitations

The strengths of this study include having a large sample size (*N* = 668). This offers practical insights on the actual implementation of HIV testing, retesting, treatment, and monitoring in Free State primary care settings, informing policy. The study included all antenatal records, regardless of the HIV status, providing a comprehensive view of the HIV prevalence, retesting, ART uptake, and neonatal outcomes among both exposed and unexposed infants. The study also identified newly diagnosed women living with HIV (22.4%), highlighting the gaps in preconception screening and early ANC, adding valuable insight for improving vertical transmission prevention from mother to child.

The limitations of the study include the fact that it was a retrospective study of maternal case records, with reliance being entirely on the quality and completeness of the records. Approximately 48.7% of the records did not include viral load. Only two primary healthcare clinics provided the data. Although selected for representativeness, the findings cannot be generalised to the entire Free State province or South Africa. There was missing information for key vertical transmission prevention indicators such as viral load, CD4 counts, and retesting of HIV. Late booking (> 20 weeks) was very common. However, reasons for the delay were unknown. Infant outcomes were only assessed at birth. Lastly, only women who attended ANC and had available records were included. Women not attending care or lost to follow-up were not captured, but may differ significantly in HIV risk or pregnancy outcomes.

### Recommendations

From the study, the following are recommended:

- Promote preconception HIV testing among couples who would like to conceive by creating awareness campaigns on adverse outcomes in infants born from mothers living with HIV.
- Promote early antenatal booking: Implement community education campaigns to raise awareness about the importance of attending ANC before 20 weeks. Strengthen collaboration with community health workers to encourage early engagement with health services.
- Strengthen HIV prevention by promoting the use of pre-exposure prophylaxis.
- Strengthen HIV monitoring and treatment: Ensure universal ART coverage for all HIV-positive women, and support adherence throughout pregnancy. Improve viral load monitoring by addressing gaps in testing and record-keeping to ensure women achieve and maintain viral suppression.
- Enhance HIV retesting practices: Enforce routine HIV retesting in line with national prevention of mother-to-child transmission (PMTCT) guidelines at specified gestational ages and during labour. Provide refresher training for healthcare workers on the importance of retesting and consistent documentation.
- Address low-birth-weight outcomes: Monitor growth and nutritional status of HIV-exposed and -unexposed infants alike. Strengthen maternal nutrition, ART adherence support, and management of pregnancy complications to reduce the risk of low birth weight.
- Strengthen health information systems: Improve completeness of maternal records, particularly for HIV viral load, obstetric complications, and follow-up investigations. Regular audits and feedback can help facilities identify and address documentation and service delivery gaps.

## Conclusion

The study demonstrates that HIV prevalence among pregnant women in the Free State province remains high, with a substantial proportion of women being newly diagnosed during pregnancy and many booking late for ANC. This limits opportunities for timely intervention. Although most HIV-positive women were initiated on ART, major gaps in monitoring, particularly the lack of viral load testing, reduce the effectiveness of prevention efforts. It is noteworthy that the inadequate retesting in HIV-negative women increases the likelihood of missed seroconversions. While no cases of mother-to-child transmission were detected, the high proportion of low-birth-weight infants, especially among HIV-exposed babies, highlights ongoing vulnerabilities in maternal and infant health. Overall, the findings highlight gaps in early antenatal booking, HIV monitoring, and retesting, as well as persistent risks for adverse neonatal outcomes.
